# Topical treatment with SPHINGOLIPIDS and GLYCOSAMINOGLYCANS for canine atopic dermatitis

**DOI:** 10.1186/s12917-020-02306-6

**Published:** 2020-03-20

**Authors:** Rosanna Marsella, Sergi Segarra, Kim Ahrens, Cristina Alonso, Lluís Ferrer

**Affiliations:** 1 Department of Small Animal Clinical Sciences, University of Florida, 2015 SW 16th Ave, Gainesville, FL 32608 USA; 2 R&D Bioiberica S.A.U, pl. Francesc Macià 7, 08029 Barcelona, Spain; 3 OWL Metabolomics, Edificio 502, Parque Tecnológico de Bizkaia, 48160 Derio, Spain; 4 Department of Medicine and Surgery, Veterinary School, Universitat Autònoma de Barcelona, Edifici V Campus UAB, 08290 Cerdanyola del Vallès, Spain

**Keywords:** Epidermal sphingolipids, Atopic dermatitis, Hyaluronic acid, Sphingomyelin, Animal model

## Abstract

**Background:**

Skin barrier dysfunction plays a key role in atopic dermatitis (AD). This impairment is related to altered composition and metabolism of epidermal sphingolipids and a deficiency of ceramides. Glycosaminoglycans (GAGs), and especially hyaluronic acid, could be useful in the management of AD. This study aimed to evaluate the effects of a novel topical treatment consisting of sphingolipids and GAGs extracts in dogs with AD. This formulation is different from previously tested products because the sphingolipid extract contained high amounts of sphingomyelin, a precursor of ceramides, and this has been shown to enhance endogenous synthesis of ceramides and to increase lamellar-related structures in vitro*.* Thus, it was hypothesized that this formulation could improve clinical disease and skin barrier function in patients with AD.

**Results:**

Twelve house dust mite (HDM) allergic atopic beagle dogs were randomized into two groups: control (*n* = 6; no treatment) or treatment (n = 6; topical sphingolipids and GAGs twice weekly for 8 weeks). Dogs were challenged with allergen twice weekly and the severity of dermatitis was scored using the canine atopic dermatitis and extent severity index (CADESI-03) once weekly. Skin barrier function (measurement of transepidermal water loss) and severity of pruritus (both pruritus visual analog scale [PVAS] and pruritus timed episodes) were assessed at 0, 4 and 8 weeks of treatment. Assessments were done by personnel unaware of group allocation. Complete blood count, serum biochemistry and *stratum corneum* (SC) lipidomics analyses were done at baseline and at week 8.

Compared to baseline, significant increases in CADESI (*P* = 0.0003) and PVAS (*P* = 0.041) were observed only in the control group, and SC polyunsaturated fatty acids increased significantly only with treatment (*P* = 0.039). Compared to control, treatment group had a significantly lower CADESI after 1 week (*P* = 0.0078) and a significantly lower PVAS after 8 weeks (*P* = 0.0448). Treatment was well tolerated.

**Conclusions:**

In this study in dogs with AD, a new topical formulation containing sphingomyelin-rich sphingolipids plus GAGs extracts attenuated the clinical worsening induced by HDM, supporting its use in atopic patients, either as an adjunctive treatment or used as monotherapy in certain cases.

## Background

Canine atopic dermatitis (AD) is a genetically predisposed inflammatory and pruritic skin disease with characteristic clinical features associated with IgE antibodies most commonly directed against environmental allergens [1]. This condition is very similar to its human counterpart in many aspects, hence the use of dogs for investigating the pathogenesis of AD as well as for testing new therapies [2, 3]. A canine model of AD in which high IgE atopic beagles are sensitized by epicutaneous application of house dust mites (HDM) [4] results in immunologic and clinical changes similar to those observed in dogs and humans with naturally-occurring AD [3]. In these dogs, flares of AD are easily triggered upon exposure to allergens. The clinical signs are progressive as long as allergen exposure is allowed.

Sphingolipids, a class of lipids containing a backbone of sphingoid bases, are essential components of animal cell membranes and have both structural and biological functions in the epidermis. The main function of epidermal sphingolipids in the *stratum corneum* (SC) of the epidermis is the formation of the skin barrier and the regulation of transepidermal water loss (TEWL). Ceramides are the main epidermal sphingolipids [5–7]. Epidermal barrier defects have been reported in both human and canine AD, leading to impaired skin barrier function. In humans, this impairment has been associated with abnormalities in epidermal lipid metabolism and lamellar body extrusion, which results in reduced ceramide levels [8–15]. Canine SC has a ceramide profile close to that of humans [16]. Reduced SC ceramide levels have also been reported in canine AD patients and associated with increased TEWL [12, 17–20]. Ceramides are derived from the hydrolysis of sphingomyelins, which is regulated by the enzyme acid sphingomyelinase [14]. In AD, sphingomyelin metabolism is altered. The enzyme sphingomyelin deacylase is highly expressed in patients with AD and competes for the common substrate (sphingomyelin) with sphingomyelinase, leading to ceramide deficiency [11, 14]. Skin barrier repair through restoration of physiological skin lipid profile has thus been suggested as a promising approach to the management of AD in both species [9, 21]. In dogs, the topical application of lipid-based formulations aimed at improving SC defects in AD has been investigated in several studies and, although they had some positive effects, they appear to be insufficient as monotherapy [21–31].

In a recent study [32] using an in vitro model of canine skin [33] the application of a unique sphingolipid extract especially rich in sphingomyelin led to increased SC ceramide levels and increased numbers of lamellar-lipid structures. Another recent in vitro study reports the ability of sphingomyelin to down-modulate PGE_2_ secretion in canine keratinocytes [34]. This extract might therefore provide clinical benefits by reducing inflammation and helping restore skin barrier function if administered in vivo.

Glycosaminoglycans (GAGs) could be useful as adjunct therapies for wound healing and maintenance of skin homeostasis. Hyaluronic acid (HA), an abundant GAG component of the skin, is involved in the wound-healing process [35, 36]. An in vitro study using human dermal fibroblasts showed increased cell proliferation and migration as well as increased hydrating capacity after application of a GAG extract with a high content of HA [37]. GAGs, and especially HA, can be useful in the management of AD [38–41]. Combining sphingolipids with GAGs has already been shown to be efficacious, as reported in humans with AD after the application of a ceramide-hyaluronic acid preparation [38, 39, 41, 42]. Furthermore, a combination of sphingolipids and GAGs has been reported to significantly enhance filaggrin expression in vitro*,* using reconstructed human epidermis [43].

The objective of this study was to evaluate the effects of a combination of the abovementioned sphingolipids [32] and GAGs [37] extracts applied topically twice weekly for 8 weeks on clinical signs and skin barrier function in atopic dogs. This formulation is different from previously tested products because the sphingolipid extract provides high amounts of sphingomyelin, a precursor of ceramides, and it has been shown to enhance endogenous synthesis of ceramides and to increase lamellar-related structures in vitro [32]. It was hypothesized that this treatment would lead to improvement in clinical signs and skin barrier function, which should be seen as a reduction in TEWL, pruritus and canine atopic dermatitis and extent severity index (CADESI)-03 scores; and an ameliorated skin lipid profile.

## Results

### Patients

Twelve house dust mite allergic atopic beagle dogs were randomized into two groups: control (*n* = 6; no treatment) or treatment (n = 6; topical sphingolipids and GAGs). At baseline, both groups were well balanced with respect to gender (3 males and 3 females per group), weight (mean ± SD: control group = 9.58 ± 1.41 kg; treatment group = 9.28 ± 0.9 kg) and age (12 months old +/− 1 week in both groups). No animals died or were euthanized during or after the study. At the completion of the study, dogs remained in the colony to be used in further studies.

### Clinical efficacy and safety endpoints

At baseline there were no significant differences (*P* > 0.05) between groups in mean CADESI, TEWL, or pruritus scores.

**Fig. 1:**
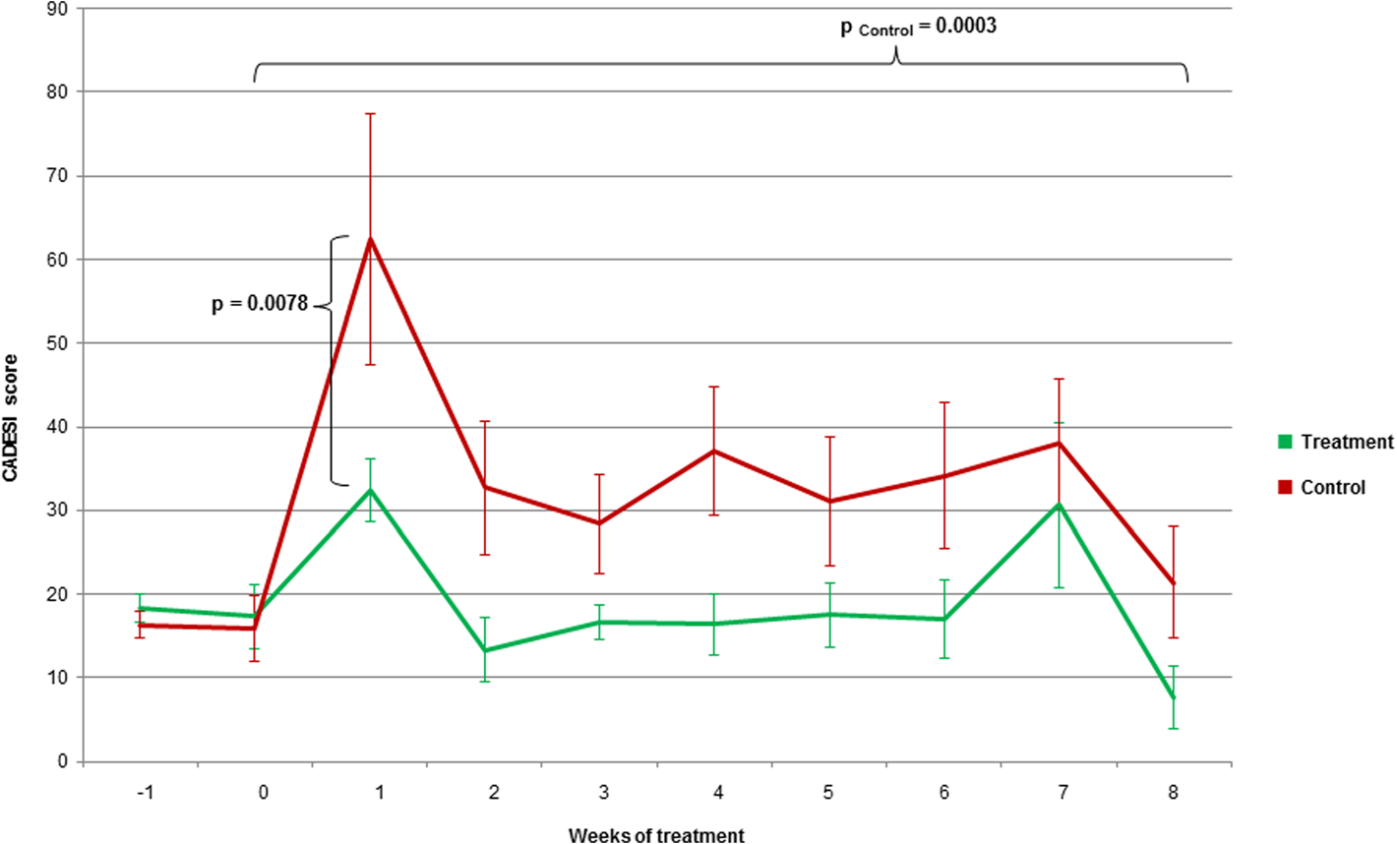
CADESI scores of AD dogs in the treatment and control groups from the study. During the study, CADESI scores were determined weekly as described in the Materials and Methods section, and are presented as the mean ± SEM (*n* = 6 vs 6)

As expected when using this canine model of AD, after 8 weeks of allergen challenge a significantly increased CADESI was observed in the control group (*P* = 0.0003) due to the allergen exposure. On the contrary, although not statistically significant, a decrease in mean CADESI was seen in the treatment group (*P* = 0.1788). When groups were compared, the treatment group presented a significantly lower mean CADESI after 1 week (*P* = 0.0078) (Fig. 1). During the rest of the study, there were no significant differences between groups.

**Fig. 2:**
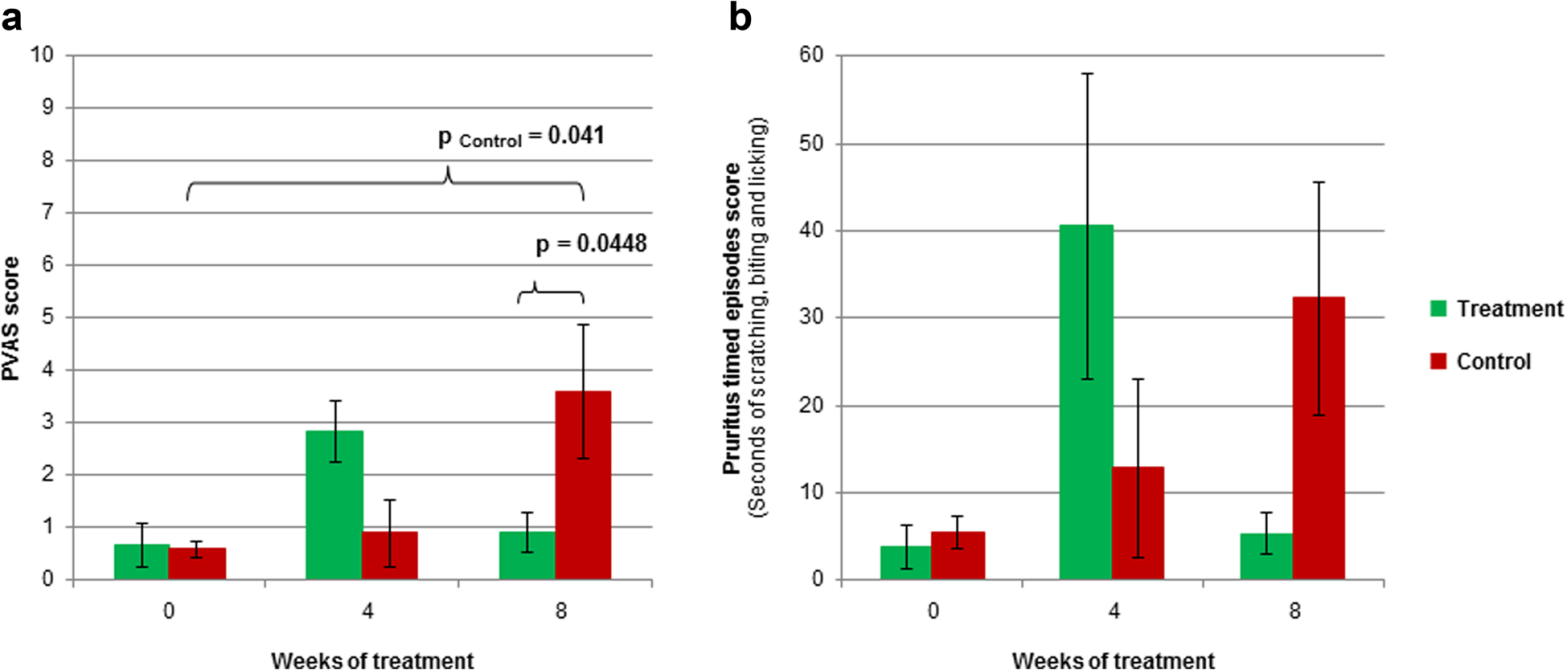
Pruritus scores in the treatment and control groups of the study. PVAS (**a**) and pruritus timed episodes (**b**) scores were evaluated at baseline and after 4 and 8 weeks of treatment. Data reported as mean ± SEM (*n* = 6 vs 6)

After 8 weeks of follow-up, a significantly increased pruritus visual analog scale (PVAS) score was seen only in the control group (*P* = 0.0414). When groups were compared, the treatment led to a significantly lower PVAS after 8 weeks (*P* = 0.0448). No significant differences were found between groups in mean pruritus timed episodes scores (Fig. 2).

**Fig. 3:**
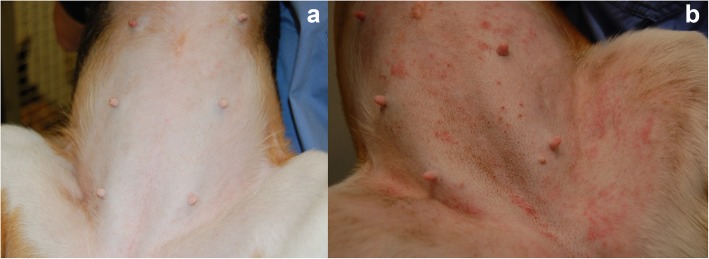
Clinical images of dogs belonging to treatment (**a**) and control (**b**) groups at the end of the study (8 weeks)

Dogs in the control group, developed clinical signs associated to AD, such as erythema, macules and papules, due to HDM challenge (Fig. 3). No abnormalities were noted in CBC or biochemistry analyses in any of the study subjects (data not shown). The administration of the topical treatment was safe and well tolerated, and no side effects were reported.

### Skin barrier function assessment

**Fig. 4:**
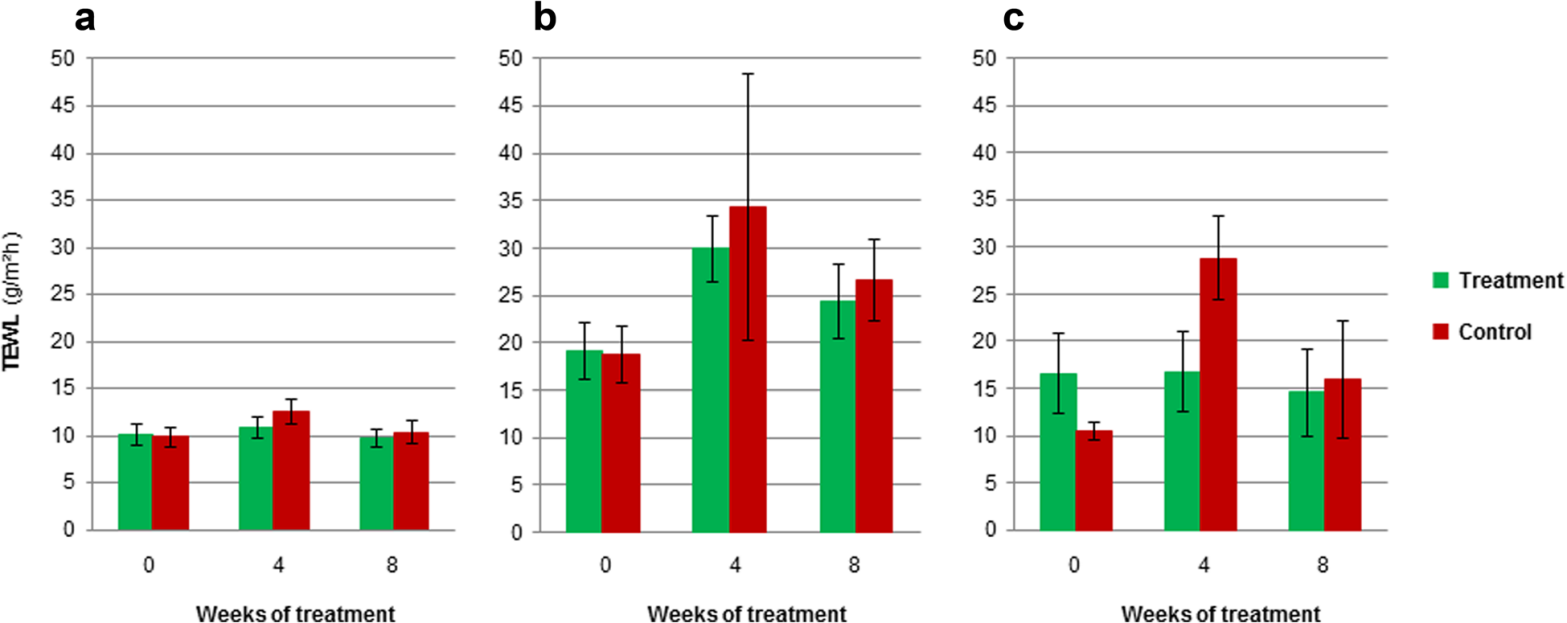
TEWL measurements for the treatment and control groups. At 0, 4 and 8 weeks of treatment, TEWL was measured from the pinnae (**a**), axilla (**b**) and inguinal area (**c**) in all AD dogs. Data reported as mean ± SEM (n = 6 vs 6)

There were no statistical significant differences in TEWL measurements in time or between groups for any of the studied body sites (Fig. 4).

**Fig. 5:**
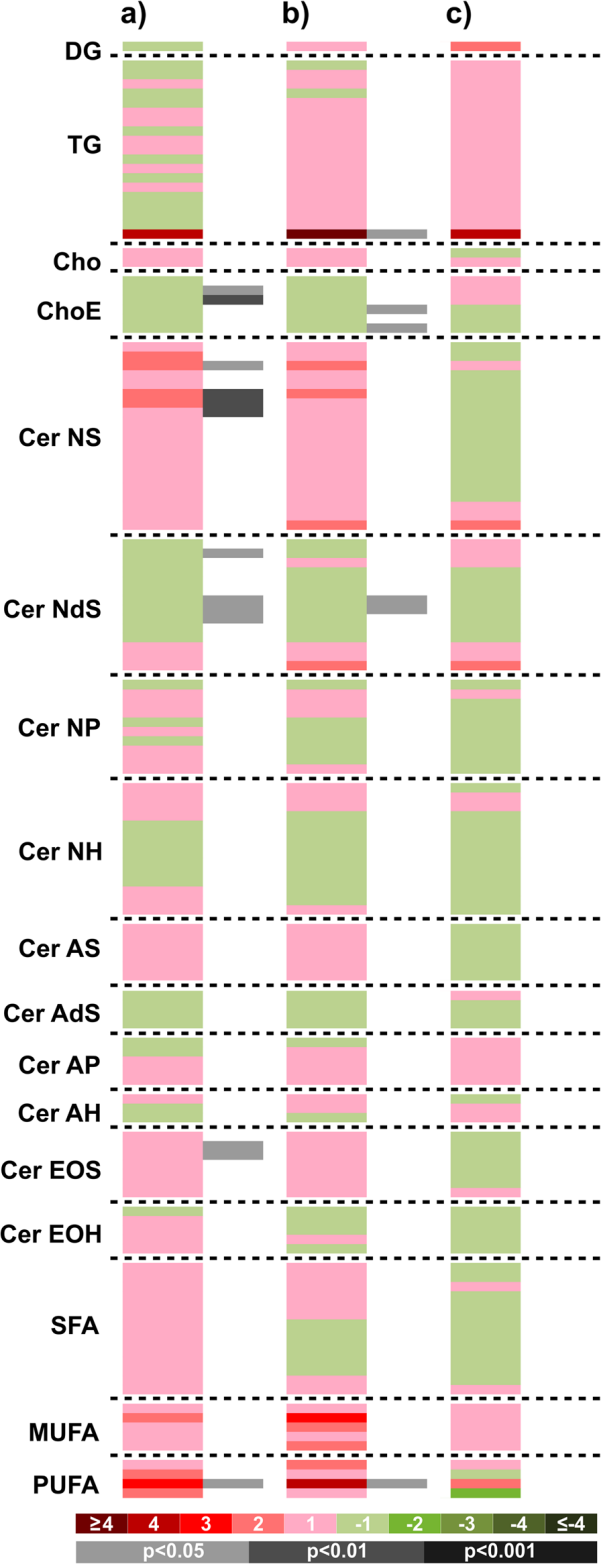
Heatmap representing binary comparisons per metabolic feature for the paired comparisons overtime in the control (**a**) and treatment (**b**) groups; and comparison treatment vs control group (**c**). Green sections of the heatmap denote metabolites that were reduced (negative log_2_ fold-changes) and red sections denote metabolites that were increased (positive log_2_ fold-changes); grey/black bars indicate significant changes with treatment (light grey, Student’s t-test p-value < 0.05; dark grey, *p* < 0.01; black, *p* < 0.001). Metabolites are grouped by families and ordered also according to their carbon number and unsaturation degree of their acyl chains

Changes in skin lipids measured from SC tape-stripping samples are shown in Table 1. Compared to baseline, a significant increase in skin polyunsaturated fatty acids (PUFAs) was seen only with the treatment (8.4 fold; *P* = 0.039), but there were no significant differences between groups (Fig. 5).

**Table 1:** Variations in lipid chemical class values in the control and the treatment groups after 8 weeks

	Control group		Treatment group	
	log2 fold-change	p value^1^	log2 fold-change	p value^1^
*Triacylglycerols*	−0.1024	0.970	0.3003	0.999
*Saturated Triacylglycerols*	−0.5770	0.066	−0.4644	0.184
*Cholesteryl Esters*	−0.5933	0.335	−0.5774	0.150
*Total Ceramides*	0.0968	0.315	−0.1821	0.298
*Ceramide NS*	0.5423	0.111	0.2131	0.944
*Ceramide NdS*	−0.4128	0.069	−0.5668	0.091
*Ceramide NP*	0.0599	0.769	−0.1532	0.284
*Ceramide NH*	−0.2078	0.208	−0.4917	0.123
*Ceramide AS*	0.4944	0.996	0.2455	0.945
*Ceramide AdS*	−0.3060	0.384	−0.1295	0.280
*Ceramide AP*	−0.0696	0.236	0.1053	0.596
*Ceramide AH*	0.0762	0.434	0.1284	0.718
*Ceramide EOS*	0.7337	0.332	0.4697	0.881
*Ceramide EOH*	0.2010	0.985	−0.0974	0.279
*Ceramide N*	0.0846	0.829	−0.2062	0.276
*Ceramide A*	0.4749	0.252	0.2258	0.855
*Ceramide EO*	0.5965	0.086	0.3288	0.852
*Saturated fatty acids*	0.0781	0.926	−0.1871	0.310
*Monounsaturated fatty acids*	0.2740	0.823	0.8698	0.419
*Polyunsaturated fatty acids*	1.6923	0.079	3.0714	0.039
*Unsaturated fatty acids*	0.7573	0.290	1.7619	0.101
*Total Non-esterified fatty acids*	0.1200	0.957	−0.0565	0.475

^1^Paired Student’s t-test

## Discussion

This study shows in vivo improvements in clinical signs, especially seen as a reduction in pruritus, after topical application of a novel treatment containing sphingolipids and GAGs (containing, mainly, sphingomyelin and HA, respectively) for 8 weeks in dogs with AD. Since an altered composition and metabolism of epidermal sphingolipids has been reported in several skin diseases including AD, new interventions –like the one tested in the present study– aimed at restoring the physiological sphingolipid metabolism may represent a convenient treatment approach [44]. Furthermore, the importance of GAGs as components of the skin as well as their reported benefits, alone or in combination with sphingolipids, makes them also a good choice for managing skin conditions [36, 37, 39, 43].

Clinically, canine and human AD are very similar [2, 3]. In the study reported herein, topical application of sphingolipids plus GAGs attenuated the worsening in CADESI 1 week after HDM application, and led to sustained lower CADESI values during the rest of the study, compared to control. Pruritus is a major clinical sign in AD [45], hence the importance of the significantly lower PVAS score observed in this study after 8 weeks of treatment. This antipruritic action could be explained by a reduced cutaneous inflammation provided by HA [36] and/or sphingomyelin [34] contained in the study treatment. Albeit not statistically significant, mean levels of pruritus were higher in the treatment group after 4 weeks, although there was a high individual variability, as it can be noted in the SEM in Fig. 2.

Short-term safety of the treatment was confirmed based on the absence of abnormalities in CBC or biochemistry analyses and on the observations that the treatment was safe and well tolerated, and no side effects were reported.

In our study, no significant differences in TEWL were found between groups for any of the studied body sites. It is important to point out that even if TEWL is commonly accepted as a non-invasive method to quickly assess skin barrier function, this method is not flawless as it can have large variability [46]. Nevertheless it was selected in this study since it is noninvasive and it has been previously shown to correlate with SC integrity [47].

Decreased amounts of ceramides in the skin of dogs with AD have been implicated in impaired barrier function of their skin [18]. It has been suggested that a decreased ceramide content accelerates TEWL in dogs and humans with AD [18, 20, 48]. Sphingomyelin is one of the most common sphingolipids found in animals [49], hence the election of this particular sphingolipid extract of animal origin for this study. This extract was chosen as it features a more suitable lipid profile for the enhancement of endogenous synthesis of ceramides [32]. Based on previous in vitro observations [32], it was expected that the application of this sphingolipid extract would lead to clinical improvements as well as an increase in epidermal ceramide levels and an amelioration in skin barrier function in vivo. In this study, lipidomics-based data from treated dogs did not show increased ceramide levels or significant differences between groups. Nevertheless, the treatment led to increased PUFAs skin levels, which could also indicate an improvement of the skin barrier. In effect, abnormalities in fatty acid profiles have been reported in AD patients, and long chain omega-3 PUFAs can potentially alter cutaneous inflammation as well as the skin epidermal barrier [23, 50, 51]. However, the complete relevance of these results remains unclear.

In recent years, several studies have been performed to assess the efficacy of different topical interventions using lipid formulations aimed at restoring the skin barrier in dogs with AD. Although a beneficial effect has been suggested for some of these therapies, current scientific evidence is still incomplete, the reported clinical improvements are modest, and none of them has yet shown a consistent effect on pruritus and skin lesions [15, 52]. Prior studies reporting the effects of topical sphingolipids have focused on the use of lipid emulsions especially rich in ceramides, showing improvements in clinical signs as well as in skin ultrastructure and lipid profiles driven by direct incorporation of these exogenous ceramides [21, 29, 30]. Although a decreased CADESI was reported after topical application of ceramides in dogs with AD in a double-blinded, randomized, controlled study [21] and in an open-label trial [25] treatment failed to achieve improvements in pruritus. Conversely, the formulation tested in this study showed a significant effect on pruritus.

It should be highlighted that the lotion used in this study contained not just ceramides but also sphingomyelin and this is different from any previously tested interventions [39, 41]. This type of sphingolipid enhances the production of endogenous skin lipids, rather than solely exerting a direct replenishment of ceramides through topical application [32]. Thus, the sphingomyelin-hyaluronic acid preparation tested in this study provides different effects and through different mechanisms of actions compared to the already existing ceramide-hyaluronic acid products [39, 41]. Additionally, this particular sphingolipid extract has been also proven to enhance the production of lamellar-related structures [32]. Unfortunately, in this study no skin biopsies were taken for electron microscopy assessment to further evaluate the effect on lipid lamellae and lamellar bodies.

The clinical benefits reported in the present study after the topical administration of sphingolipids and GAGs, especially on pruritus, confirm the efficacy of such combination. It should be stressed that dogs were not on any other medication, which indicates a remarkable benefit of this topical treatment as a sole therapy. It is, therefore, reasonable to speculate that some naturally-occurring cases of canine AD, especially young patients that have been diagnosed at very initial stages, could benefit from this treatment alone. Besides that, if used in combination with other therapies, it might result in faster improvements, allow a reduction in the need and dosage of other drugs and be useful for the long-term management of such chronic condition.

Our findings were obtained using a validated canine model of AD worsened by HDM exposure [4]. Clinically, dogs in the control group behaved as expected when using this model. Whether animals can be used to predict human response to certain treatments is based on similarities between species and does not always correlate completely yet dogs are a better model for people than any other species as their disease shares the complexity of the human condition. In the authors’ opinion, although direct extrapolations between species should obviously be made with caution, data presented herein indicates a beneficial effect of this topical combination in dogs with AD but also suggests its potential application in atopic people as part of the multimodal treatment approach since dogs appear to respond similarly to therapy for AD as humans.

This study has some limitations which should be pointed out. First, the sample size was small. Since this study was the first in vivo experimental approach to assessing the effects of this novel intervention, it was expected that six animals per group would provide enough statistical power. Second, in the herein study, SC function evaluation using TEWL showed no differences between study groups. Perhaps simultaneous TEWL and SC water content measurements could have provided a more detailed characterization of the skin function [53]. Moreover, no skin biopsies were taken from the dogs in this study. Histopathological evaluation of skin samples could have provided more valuable information on the effects of the intervention. Further studies involving a more thorough characterization of the changes produced in the skin after treatment and using a larger number of study subjects are therefore warranted. On the other hand, given that this study only evaluated short-term safety based on CBC and biochemistry, local safety of the product in the skin, both short- and long-term, should also be assessed. Since the intervention attenuated the clinical worsening due to HDM application, the effects of this treatment should also be evaluated in canine patients presenting with already established clinical signs associated with AD. Studies evaluating the effects of GAGs or sphingolipids separately are also needed. Lastly, despite the evidence that supports the use of the dog as an adequate animal model of human AD, it would be necessary to perform clinical studies in people to evaluate the effects of this treatment in human AD patients.

## Conclusions

In this study in dogs with AD, a topical combination of sphingolipids and GAGs attenuated the clinical worsening induced by HDM, supporting the use of this treatment in atopic patients, either as an adjunctive treatment or used as monotherapy in certain cases.

## Methods

All procedures used in this study were reviewed and approved by the Institutional Animal Care and Use Committee of the University of Florida (reference number: 201508927). The study was conducted according to the NIH Guide for the Care and Use of Laboratory Animals.

### Animals and housing

Animals included in the study belonged to a research colony of allergic atopic beagles from the Department of Small Animal Clinical Sciences at the University of Florida (Gainesville, FL, USA). Dogs had to be healthy based on physical examination, and had to be clear of clinical signs of pyoderma at the time of enrollment. During the study, dogs were housed in an Institutional Animal Care and Use Committee (IACUC)-monitored facility of the School of Veterinary Medicine at the University of Florida (Gainesville, FL, USA). All animals were fed the same diet (Science Diet® Small bites, Hill’s Pet Nutrition, Inc., Topeka, KS, USA) and received no concomitant therapies during the conduct of this study. Dogs were epicutaneously challenged with HDM administration into the inguinal area. HDM were prepared from culture (natural *Dermatophagoides farinae*, Greer Laboratories Inc., Lenoir, NC, USA) and mixed with phosphate buffered saline (PBS; pH 7.2) to a final concentration of 15.6 mg/mL. The HDM solution (1.6 mL, 25 mg/dog/challenge) was applied twice weekly for 8 weeks.

### Study design

This study was designed as a prospective, double-blinded, controlled clinical trial with two parallel arms. Dogs were randomly allocated to the control (*n* = 6) or the treatment (n = 6) groups, using an assignment of numbers to each dog and blind hat draw. Dogs in the control group did not receive any intervention. Dogs in the treatment group were administered a topical product (Atopivet® Spot-on, Bioberica S.A.U., Barcelona, Spain). This lotion contains 0.5% of a sphingomyelin-rich sphingolipid extract (Biosfeen®, Bioiberica S.A.U., Barcelona, Spain) and 0.5% of a HA-rich GAGs extract (Dermial®, Bioiberica S.A.U., Barcelona, Spain) as active ingredients. The product was applied topically, twice weekly for 8 weeks, on the pinnae, axilla, interdigital areas of front and back paws, inguinal area, chest, dorsum (between the shoulder blades and several more spots further back), a drop in each area (one mono-dose pipette of 2 mL per dog). The product and the HDM challenge were applied on the same weekdays. The topical treatment was administered 2 hours after allergen application in order to simulate a situation in which inflammation had been already triggered so that the intervention was used as treatment rather than prevention.

### Clinical evaluations

All dogs were evaluated using a validated scoring system CADESI-03 at baseline and weekly until the end of the study (8 weeks). These evaluations were all performed by the same investigator (RM), who was blinded to treatment allocation.

Pruritus was assessed at baseline and after 4 and 8 weeks of treatment, using two different methods: pruritus timed episodes and global pruritus scoring. Pruritus timed episodes were evaluated during 20 min for licking, scratching and biting. Each episode was recorded as a timed period of seconds of licking, scratching and biting. A global pruritus score was subjectively assigned to each dog after the 20-min timed episode pruritus evaluation using a PVAS modified from a score by Hill et al. [54], ranging from normal (0) to very severe itching [10]. Marks were made on the PVAS and subsequently measured using a 10-point transparency placed over the scale, and recorded. All investigators involved in clinical evaluations were blinded to treatment assignment, which was administered by independent personnel.

### Blood sampling

Before and after treatment, 4 mL of blood were drawn from each dog by jugular venipuncture, and divided into serum and EDTA tubes to measure changes in CBC and biochemistry. These analyses were performed at the University of Florida, Veterinary Hospital, clinical pathology laboratory (Gainesville, FL, USA).

### TEWL measurements

TEWL was measured at baseline and after 4 and 8 weeks of treatment at three different anatomical sites (pinnae, axilla and inguinal area) in triplicate. Dogs were acclimated for 30 min in a humidity controlled room prior to taking measurements. Measurements involved the application of the probe of the close chamber evaporimeter (VapoMeter®, Delfin Technologies Ltd., Kuopio, Finland). Readings measured as an increase in relative humidity inside the closed chamber over a set time in g/m^2^h were obtained after 10 s of close contact with the skin and wirelessly transmitted to a laptop. Means and standard deviation for the readings were calculated for each site evaluated.

### Tape stripping

Lipid composition in the SC was assessed by tape-stripping at baseline and after 8 weeks of treatment. One day prior to obtaining the sample, the hair in the area of non-lesional inguinal skin was shaved. Gloves were used during the handling of the tape strips and the contact zone of the adhesive was avoided with fingers or other material. First, two D-Squame adhesive tapes (D-Squame, size 22 mm, CuDerm Corporation, Detroit, MI, USA) were applied one time each one, using uniform pressure, and discarded. After that, one D-Squame adhesive tape (size 22 mm) was applied 10 times using uniform pressure in alternating directions. The tape strip was immediately placed into an appropriately labeled tube (one tape strip per tube), tightly sealed and avoiding direct contact of the adhesive zone with the plastic. Tubes were then placed on dry ice and transferred to a − 80 °C freezer. At the end of the study, pre- and post-treatment tape-stripping samples were transported on dry ice and with temperature control log to a specialized lab (OWL Metabolomics, Derio, Spain) for lipidomics analysis.

### Lipidomics analysis

Lipidomics analyses were performed by OWL Metabolomics (Derio, Spain). Two ultra-high performance liquid chromatography coupled to time-of-flight mass spectrometry (UHPLC-ToF-MS)-based platforms were used for optimal profiling of the SC lipidome: Platform 1 was used to analyze fatty acids (FA) while glycerolipids, cholesteryl esters, and sphingolipids where analyzed in Platform 2 as previously described [55] (see Additional file 1).

For protein quantification, the Squamescan 850A (Heiland Electronic, Wetzlar, Germany) was used to determine the amount of SC removed to obtain a good indication of the depth of each tape strip taken, measuring the protein content.

All data were processed using the TargetLynx application manager for MassLynx 4.1 software (Waters Corp.) as previously described by Martínez-Arranz et al. [56]. The peak detection process included 139 LC–MS features.

Normalization factors were calculated following the procedure described [56]. Further normalization procedure was applied by dividing every sample by its protein content, as part of the biological normalization.

### Statistical analysis

No formal sample size calculation was performed. The number of dogs used in the study was dictated by the size of the colony. However, it was considered that 12 dogs would be enough given the homogeneity of the study subjects and the fact that they share housing conditions, which should reduce variability among them. To evaluate differences at the multiple times during the intervention a linear mixed effects models was run with CADESI, pruritus, or TEWL as outcomes, the corresponding baseline measure as the covariate, and day/month, intervention indicator, and the interaction between the two as the predictors. A random subject-specific intercept was included to account for the within-subject correlation. Model-based tests were conducted for overall within group temporal change, overall group difference between the two groups, and pairwise group difference between the groups at each observed time point. *P* < 0.05 was considered significant. Post-hoc calculations revealed that 15 dogs per group would have been needed to provide enough statistical power.

For the lipidomic analysis, univariate statistical analyses were performed in order to evaluate the effect of the treatment, calculating group percentage changes and paired Student’s t-test *p*-value (or Welch’s t test where unequal variances were found), comparing initial and final values in each group. All calculations were performed using statistical software package R v.3.1.1 (R Development Core Team, 2011; http://cran.r-project.org).

## Supplementary information

**Additional file 1.** Lipidomics analysis. This additional file explains the lipidomics analysis in more detail.

## Data Availability

The datasets used and/or analyzed during the current study available from the corresponding author on reasonable request.

## Abbreviations

AD: Atopic dermatitis
CADESI: Canine atopic dermatitis and extent severity index
FA: Fatty acids
GAGs: Glycosaminoglycans
HA: Hyaluronic acid
HDM: House dust mites
IACUC: Institutional Animal Care and Use Committee
PUFAs: Polyunsaturated fatty acids
PVAS: Pruritus visual analog scale
SC: *Stratum corneum*
TEWL: Transepidermal water loss
UHPLC-ToF-MS: Ultra-high performance liquid chromatography coupled to time-of-flight mass spectrometry
