# Supplementary material for: Topical treatment with SPHINGOLIPIDS and GLYCOSAMINOGLYCANS for canine atopic dermatitis
Source: BMC Vet Res. 2020 Mar 20;16:92. doi: 10.1186/s12917-020-02306-6 (PMC7082980; doi:10.1186/s12917-020-02306-6)
Supplement: Supplementary file 1 — Additional file 1. Lipidomics analysis. This additional file explains the lipidomics analysis in more detail. [file 12917_2020_2306_MOESM1_ESM.docx]

**Additional file 1**

**Lipidomics analysis**

Lipidomics analyses were performed by OWL Metabolomics (Derio, Spain). Two ultra-high performance liquid chromatography coupled to time-of-flight mass spectrometry (UHPLC-ToF-MS)-based platforms were used for optimal profiling of the SC lipidome. 800 µL of chloroform/methanol (2:1, v/v) were added to every tape and were homogenized at 6500 rpm for 23 seconds in a Precellys 24 homogenizer (Bertin Technologies, Orléans, France). This extraction solvent was spiked with lipids not detected in unspiked SC extracts: NEFA(19:0) and NEFA(13:0) were used as internal standards in the first UHPLC-MS analysis (Platform 1), while sphingomyelins (SM) (d18:1/6:0), phosphatidylethanolamine (PE) (17:0/17:0), triacylglycerols (TAG) (13:0/13:0/13:0), ceramides (Cer) (d18:1/17:0) and cholesteryl esters (ChoE) (12:0) were used in the second analysis (Platform 2). Samples were kept at -20 ºC for 30 minutes and were centrifuged at 18,000 x g at 4 ºC for 10 minutes. Each well in an OSTRO plate (Waters Corp., Milford, MA, USA) was conditioned 5 times with 800 µL of methanol. Subsequently, 700 µL of sample supernatant were loaded, followed by 800 µL of methanol. Both eluents were collected together and evaporated under vacuum at 60 ºC for 50 min. Samples were resuspended in 100 µL of acetonitrile/isopropanol (1:1, v/v) and shaken for 10 minutes. Samples were centrifuged for 10 min at 4 ºC and 18,000 x g and finally supernatants were collected to be analyzed in each UHPLC-MS platform.

Platform 1 was used to analyze fatty acids (FA). Extracts (3 µl) were then introduced into the ACQUITY UPLC® system (Waters Corp.) with a 2.1×100mm ACQUITY 1.7 µm C18 BEH column (Waters Corp.) maintained at 40ºC. Chromatographic flow rate was 0.4 ml/min, for a total run time of 18 min. The following linear elution gradient was used: 90% solvent A (water, acetonitrile, and 10mM ammonium formate), to which solvent B (acetonitrile, isopropanol, and 10mM ammonium formate) was added incrementally to reach a concentration of 20% B after 3 min, and sequentially 40% B until 5 min, 45% B until 5.5 min, 80% B until 12 min, 100% B until 12.1 min and returning to the initial composition at 15 min, at which it was maintained for a further 2 min. Analysis was performed using the aforementioned UHPLC system coupled online to a Waters Xevo G2 QToF (Waters Corp.) with electrospray ionization. Capillary and cone voltages were set in negative ion mode at 2800V and 50V, respectively. The nebulizer N2 gas was set at a flow rate of 1000 l/h and 500˚C, cone gas at 30 L/h and the source temperature of 120˚C.

Platform 2: Glycerolipids, cholesteryl esters, and sphingolipids UHPLC-MS profiling

Diacylglycerols (DAG), TAG, ChoE, cholesterol, cholesteryl sulfate, SM, and Cer were analyzed in this analytical platform. The analysis was performed using the ACQUITY UPLC® system (Waters Corp.) coupled online to a Waters Xevo G2 QToF (Waters Corp.) with a 2.1×100mm ACQUITY 1.7 µm C18 BEH column (Waters Corp.) maintained at 60˚C. Sample extracts (3 µL) were injected onto the column and eluted at a flow rate of 0.4 ml/min with a total run time of 17 min. The mobile phase consisted of solvent A (water, acetonitrile, and 10mM ammonium formate) and solvent B (acetonitrile, isopropanol, and 10mM ammonium formate) and the following elution gradient was used: 40% solvent B, increasing linearly to 100% over 10 min and returning to the initial composition over 5 min, at which it was maintained for a further 2 min. Mass spectrometry was used in positive ion modes with the capillary current set at 3200V and the cone voltage at 30V. The nebulizer gas was set at a flow rate of 1000 l/h and 500˚C, the cone gas at a flow rate of 30 l/h and a source temperature of 120˚C.

For protein quantification, the Squamescan 850A (Heiland Electronic, Wetzlar, Germany) was used to determine the amount of SC removed to obtain a good indication of the depth of each tape strip taken, measuring the protein content.

All data were processed using the TargetLynx application manager for MassLynx 4.1 software (Waters Corp.). A set of predefined retention time, mass-to-charge ratio pairs, Rt-m/z, corresponding to metabolites included in the analysis are fed into the program. Associated extracted ion chromatograms (mass tolerance window = 0.05 Da) are then peak-detected and noise-reduced in both the LC and MS domains such that only true metabolite related features are processed by the software. A list of chromatographic peak areas is then generated for each sample injection. The peak detection process included 139 LC–MS features, identified prior to the analysis. A moving average smoothing method was applied for noise reduction; this is a technique that removes random noise from measured LC–MS signals.

Normalization factors were calculated for each metabolite by dividing their intensities in each sample by the recorded intensity of an appropriate internal standard in that same sample, following the procedure described by Martínez-Arranz et al. (Martinez-Arranz I, Mayo R, Perez-Cormenzana M, Mincholé I, Salazar L, Alonso C, Mato JM. Enhancing metabolomics research through data mining. J. Proteomics 2015;127(B)275-88). Further normalization procedure was applied by dividing every sample by its protein content, as part of the biological normalization.
